# The Clinical Features and Immunological Signature of *Cyclospora cayetanensis* Co-Infection among People Living with HIV in Ghana

**DOI:** 10.3390/microorganisms10071407

**Published:** 2022-07-13

**Authors:** Fred Stephen Sarfo, Albert Dompreh, Shadrack Osei Asibey, Richard Boateng, Felix Weinreich, Edmund Osei Kuffour, Betty Norman, Veronica Di Cristanziano, Hagen Frickmann, Torsten Feldt, Kirsten Alexandra Eberhardt

**Affiliations:** 1 Department of Medicine, Kwame Nkrumah University of Science and Technology, Kumasi 00233, Ghana; stephensarfo78@gmail.com (F.S.S.); branorman@yahoo.com (B.N.); 2 Department of Medicine, Komfo Anokye Teaching Hospital, Kumasi 00233, Ghana; shakosbey19@gmail.com; 3 Department of Clinical Microbiology, Komfo Anokye Teaching Hospital, Kumasi 00233, Ghana; adompreh@gmail.com (A.D.); richardboateng166@gmail.com (R.B.); 4 Department of Microbiology and Hospital Hygiene, Bundeswehr Hospital Hamburg, 20359 Hamburg, Germany; felixweinreich@bundeswehr.org (F.W.); frickmann@bnitm.de (H.F.); 5 Laboratory of Retrovirology, The Rockefeller University, New York, NY 10065, USA; eosei@rockefeller.edu; 6 Institute of Virology, Faculty of Medicine and University Hospital Cologne, University of Cologne, 50937 Cologne, Germany; veronica.di-cristanziano@uk-koeln.de; 7 Institute for Medical Microbiology, Virology and Hygiene, University Medicine Rostock, 18057 Rostock, Germany; 8 Clinic of Gastroenterology, Hepatology and Infectious Diseases, University Hospital Düsseldorf, 40225 Düsseldorf, Germany; torsten.feldt@med.uni-duesseldorf.de; 9 Department of Tropical Medicine, Bernhard Nocht Institute for Tropical Medicine & I. Department of Medicine, University Medical Center Hamburg-Eppendorf, 20359 Hamburg, Germany; 10 Division of Hygiene and Infectious Diseases, Institute of Hygiene and Environment, 20539 Hamburg, Germany

**Keywords:** parasite, immunodeficiency, diarrhea, Sub-Sahara, epidemiology, Africa, enteric infection, cyclosporiasis

## Abstract

Background: There is a paucity of information on the contemporary burden, disease patterns, and immunological profile of people living with HIV who are co-infected with *C. cayetanensis* in the post-antiretroviral therapy era. Methods: For this cross-sectional study, stool samples of 640 HIV-positive and 83 HIV-negative individuals in Ghana were tested for *C. cayetanensis*. Additionally, sociodemographic parameters, clinical symptoms, medical drug intake, and immunological parameters were assessed. Results: The prevalence of *C. cayetanensis* was 8.75% (*n* = 56) in HIV-positive and 1.20% (*n* = 1) in HIV-negative participants (*p* = 0.015). Within the group of HIV-positive participants, the prevalence reached 13.6% in patients with CD4+ T cell counts below 200 cells/µl. Frequencies of the clinical manifestations of weight loss and diarrheal disease were significantly higher in patients with *C. cayetanensis* compared to those without co-infection (36.36% vs. 22.59%, *p* = 0.034 and 20.00% vs. 4.90%, *p* < 0.001, respectively). The expression of markers of immune activation and exhaustion of T lymphocyte sub-populations was significantly elevated in patients colonized with *C. cayetanensis*. Conclusions: In the modern post-combined antiretroviral therapy (cART) era, the acquisition of *C. cayetanensis* among PLWH in Ghana is driven largely by the immunosuppression profile characterized by high expression of markers of immune activation and immune exhaustion.

## 1. Introduction

*Cyclospora cayetanensis*, a coccidian parasite, causes enteric disease among humans via fecal–oral transmission [1,2]. The life cycle of this parasite involves an obligatory sporulation step at a temperature of 25–30 °C for at least 1–2 weeks in the environment to become infective [3]. Therefore, direct person-to-person transmission is rather unlikely [2]. Infection with *C. cayetanensis* is mainly transmitted through the ingestion of contaminated food or water with oocysts and heightened transmission during the rainy season [4,5,6]. *C. cayetanensis* establishes as an infection in the upper small intestinal tract, which is evanescent in immunocompetent hosts, but among immunocompromised individuals, complications that may ensue include chronic diarrhea and malabsorptive syndromes due to villous atrophy and crypt hyperplasia [7]. These characteristics of *C. cayetanensis* make it an important pathogen among people living with HIV (PLWH) as a differential diagnosis of chronic diarrheal disease in this population.

Infection with *Cyclospora cayetanensis* is treated with co-trimoxazole, a commonly prescribed antibiotic used prophylactically and therapeutically among PLWH in resource-limited settings [8,9]. Ciprofloxacin is less effective than co-trimoxazole but is suitable for patients who are intolerant to sulfonamide drugs [10].

There is, however, a paucity of information on the contemporary burden, disease patterns, and immunological profile of PLWH who are co-infected with *C. cayetanensis* in the post-antiretroviral therapy era. Recent reviews confirmed diarrhea and a low CD4+ T cell count to be significantly associated with *C. cayetanensis* infection in PLWH [3,11]. We, therefore, sought to conduct a study to evaluate the clinical and immunological profile of a large cohort of PLWH according to infection status with *C. cayetanensis* in a Ghanaian health system. Furthermore, we aimed to provide new information on the association of this parasite with markers of immune activation, cell proliferation, terminal differentiation, and exhaustion of CD4+ and CD8+ T lymphocyte sub-populations stratified for the combined antiretroviral therapy (cART) status.

## 2. Materials and Methods

### 2.1. Study Design and Population

This cross-sectional observational study is part of a work investigating the prevalence of *Helicobacter pylori* and other gastrointestinal co-infections in HIV-positive and -negative adults and was conducted at the Komfo Anokye Teaching Hospital, a tertiary referral hospital in the Ashanti Region of Ghana [12,13,14,15]. Between November 2011 and November 2012, consecutive HIV-positive patients presenting to the HIV outpatient department, and HIV-negative blood donors presenting to the blood bank of the hospital, were offered participation in the study. All participants gave written informed consent prior to enrolment. The study was approved by the appropriate ethics committees in Ghana (CHRPE/AP/12/11) and Germany (PV3771).

### 2.2. Data Collection and Laboratory Methods

Demographic, socioeconomic, and clinical data, as well as a detailed medical history, were recorded using standardized questionnaires, which were completed by trained study personnel. In particular, time since diagnosis of HIV infection, duration and kind of cART, co-medications, and clinical parameters were documented. Blood samples were collected, and the analysis of CD4+ T cell count was performed locally using a FACSCalibur flow cytometer (Becton Dickinson, Mountain View, CA, USA). HIV-1 viral load was measured using the Real-Time HIV-1 PCR system (Abbott Diagnostics, Wiesbaden, Germany).

Peripheral blood mononuclear cells (PBMCs) were isolated by centrifugation of heparinized venous blood on a Ficoll/Hypaque (Biocoll Separating Solution, Biochrom AG, Berlin, Germany) density gradient. Cells were washed in phosphate-buffered saline and resuspended in Roswell Park Memorial Institute 1640 medium (both Gibco Invitrogen, Carlsbad, CA, USA) supplemented with heat-inactivated fetal calf serum (Biochrom AG, Berlin, Germany). PBMCs were cryopreserved and shipped to Germany on liquid nitrogen. Cell surface markers for immune activation were stained as described elsewhere [16]. Flow cytometric data were acquired using the LSRII flow cytometer (BD Biosciences, Heidelberg, Germany) and analyzed using FlowJo version 9.6.2 (Tree Star, San Carlos, CA, USA).

Aliquots of native stool samples were freshly frozen and stored at −80 °C before being transported to Germany on dry ice. All stool samples were subjected to nucleic acid extraction applying the QiaAMP DNA Stool Mini kit (Qiagen, Hilden, Germany) as suggested by the manufacturer. The real-time PCR targeting the small subunit ribosomal RNA (SSU rRNA) gene of *C. cayetanensis* was run as described previously on a RotorGene Q cycler (Qiagen, Hilden, Germany) with some minor modifications [17]. In short, the forward primer sequence was 5′-TAGTAACCGAACGGATCGCATT-3′, the reverse primer sequence was 5′-AATGCCACGGTAGGCCAATA-3′, and the probe sequence was 5′-CCGGCGATAGATCATTCAAGTTTCTGACC-3′. The reaction mix comprised a 10 μL HotStar master mix (Qiagen, Hilden, Germany), 5.0 mM total MgCl_2_, and 2.0 μL DNA eluate in 20 µL volumes. The run conditions were activation at 95 °C for 15 min, 45 cycles of 15 s denaturation at 95 °C, followed by a touchdown for the 30-second-long combined annealing and elongation step from 72 °C to 67 °C over 13 cycles in 0.5 °C steps, with final cooling down to 40 °C for an additional 30 s at the end of the run. As estimated in previous studies, calculated sensitivity for the *C. cayetanensis*-specific real-time PCR ranges from 32.0% to 81.8%, specificity from 98.7% to 99.7% with a limit of detection of less than 10 copies per µL eluate [18,19]. All runs included a PCR-grade-water-based negative control and positive control with a plasmid containing the target sequence 5′-GATTCATAGTAACCGAACGGATCGCATTTGGCTTTAGCCGGCGATAGATCATTCAAGTTTCTGACCTATCAGCTTTCGACGGTAGGGTATTGGCCTACCGTGGCATTGACGGG-3′. An inhibition control PCR targeting a phocid herpes virus (PhHV) sequence fragment as described elsewhere was run with all samples to exclude relevant sample inhibition [20].

### 2.3. Statistical Analysis

Categorical variables were compared using either the χ^2^ test or the Fisher exact test, as appropriate. Continuous variables were expressed as median (interquartile range, IQR) or mean ± standard deviation (SD) and compared using the Wilcoxon rank-sum test or the unpaired Student’s *t*-test. Multiple regression models were accomplished using the “forestmodel” package in R (version 4.0.5, R Foundation for Statistical Computing, Vienna, Austria). To account for moderation, models were additionally run with included interaction terms. The Spearman rank correlation coefficient ρ was calculated as a measure of the strength of the relationship between continuous variables. Two-sided *p*-values were presented, and an α of 0.05 was determined as the cutoff for significance.

## 3. Results

### 3.1. Composition of the Study Population

**Figure 1:**
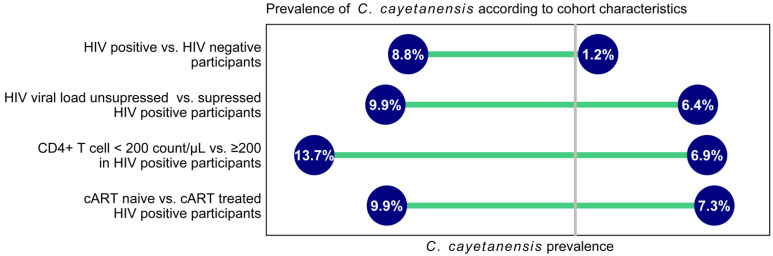
Prevalence of *C. cayetanensis* according to cohort characteristics. cART—combined antiretroviral therapy.

A total of 1095 HIV-positive individuals and 107 HIV-negative blood donors were recruited for this study. Residual stool samples for *C. cayetanensis* testing were available for 723 individuals (640 HIV-positive and 83 HIV-negative). The prevalence of *C. cayetanensis* was 8.75% (*n =* 56) in HIV-positive participants and 1.20% (*n =* 1, *p =* 0.015) in HIV-negative participants. Among HIV-positive patients, the detection rate was higher in patients with detectable HIV viral load, with CD4+ T cell counts below 200/µL, and in cART naïve patients (Figure 1).

### 3.2. Comparison of Demographic and Clinical Characteristics of the HIV Cohort by C. cayetanesis Status

As shown in Table 1, there were no differences in the mean age, sex, and socioeconomic parameters of those infected with *C. cayetanensis* compared to those who were uninfected. There were also no differences in the proportion of cART and co-trimoxazole chemoprophylaxis between the two groups, but those with *C. cayetanensis* infection had been on cART for a significantly shorter median (IQR) duration of 17.3 (6.9–36.6) months compared to 55.2 (28.6–79.5) months for those not co-infected (*p =* 0.001).

**Figure 2:**
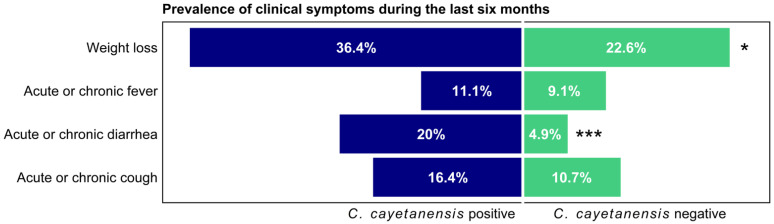
Prevalence of clinical symptoms during the last six months according to *C. cayetanensis* status among HIV-positive participants. * *p* < 0.05; *** *p* < 0.001.

Clinically, those with *C. cayetanensis* co-infection compared to those without co-infection were more likely to report weight loss over the previous 6 months (36.36% vs. 22.59%, *p =* 0.034) and acute or chronic diarrhea (20.00% vs. 4.90%, *p* < 0.001), as shown in Figure 2. There were no significant differences in other symptoms such as fever or chronic cough.

### 3.3. Comparison of Virological and Immunological Characteristics of the HIV Cohort by C. cayetanesis Status

Participants with *C. cayetanensis* co-infection had a significantly higher median HIV-1 viral load in log10 copies/mL (5.0 [2.2–5.6 IQR] vs. 4.1 [1.6–5.3 IQR], *p* < 0.012, Table 2) and a correspondingly lower CD4+ T cell count/µL (228.0 [91.5–395.5 IQR] vs. 355.0 [163.5–586.5 IQR], *p =* 0.001) than those without this co-infection. Additionally, when stratified for cART exposure, the CD4+ T cell count was lower in *C. cayetanensis* carriers (308 [260–600 IQR] vs. 517 [329–713 IQR], *p =* 0.053 and 141 [46–298 IQR] vs. 246 [94–451 IQR], *p* < 0.001, respectively). The CD4+/CD8+ T cell ratio, which is inversely associated with immune activation in HIV, was significantly lower in *C. cayetanensis* carriers (0.3 [0.1–0.4 IQR] vs. 0.4 [0.2–0.7 IQR], *p =* 0.005). HIV positive individuals with *C. cayetanensis* co-infection had a significantly higher expression of HLA-DR+CD38+ on CD4+ and CD8+ T lymphocytes as additional markers of immune activation (31.0 [19.2–38.6 IQR] vs. 17.3 [9.5–30.6 IQR], *p =* 0.002 and 49.3 [42.8–60.9 IQR] vs. 43.4 [27.8–56.7 IQR], *p =* 0.042, respectively). Furthermore, markers of immune exhaustion (PD-1+) on CD8+ T lymphocytes were elevated in individuals with detected *C. cayetanensis* compared to those without this pathogen (43.1 [30.1–49.2 IQR] vs. 31.9 [21.4–44.0], *p =* 0.042). There were no significant differences in the expression of surface markers for terminal differentiation or cell proliferation in *C. cayetanensis* positive and negative patients.

### 3.4. Immunological Significance of C. cayetanensis Infection in PLWH

In a multiple logistic regression model, we identified a CD4+ T lymphocyte value < 200 cells/µL to be significantly associated with the odds of *C. cayetanensis* co-infection among PLWH, the adjusted odds ratio was 2.11 (95% CI: 1.17, 3.79), *p =* 0.01 (Supplementary Figure S1a). However, age, sex, and co-trimoxazole use were not associated with *C. cayetanensis* infection. Furthermore, *C. cayetanensis* infection among PLWH was significantly and independently associated with markers of increased immune activation with a parameter estimate from a multiple linear regression model of 7.46 (95% CI: 1.65, 13.27, *p =* 0.01). As expected, a low CD4+ T lymphocyte count of <200/µL also was associated with elevated immune activation, as evidenced by increased expression of HLA-DR+ CD38+ CD4+ (Supplementary Figure S1b).

### 3.5. Correlations of Cycle Threshold (Ct) Values with HIV Viral Load and CD4+ Cell Count

The correlation analysis of cycle threshold (Ct) values and HIV viral load as well as CD4+ T cell count in *C. cayetanensis* positive participants revealed a significant inverse correlation for the HIV viral load and the Ct values of *C. cayetanensis* but not for the CD4 lymphocyte count (*rho* = −0.36, *p =* 0.008 and *rho* = 0.10, *p =* 0.469, respectively).

## 4. Discussion

In this large cohort of Ghanaians living with HIV in the post-cART era, the prevalence of *Cyclospora cayetanensis* was 8.75% compared to 1.20% among HIV-negative control group. Compared with data from other regions of the globe, the prevalence of *C. cayetanensis* among the PLWH in Ghana is higher than 0.3% in Iran, 1% in Tanzania, 1.1% in Thailand, 0.7–3.3% in India, 3.0% in Cuba, 3.4% in Nigeria, and 3.9% in Venezuela but lower than 11% in a Haitian cohort [21,22,23,24,25,26,27]. The differences in prevalence observed in the various studies may reflect differences in relative proportions of cART exposure in the HIV cohort, diagnostic techniques utilized (microscopy or molecular approaches such as PCR), prophylactic use of co-trimoxazole and perhaps also the level of sanitation in the various countries where these studies were conducted. In the present study, the presence of *C. cayetanensis* was assessed using an in-house real-time PCR targeting the small subunit ribosomal RNA (SSU rRNA) gene of *C. cayetanensis* [19]. In a previous head-to-head-comparison of described *C. cayetanensis*-specific real-time PCR assays, the applied approach was confirmed to be particularly sensitive for the identification of *C. cayetanensis* in human stool samples [19].

In our cohort, the prevalence of *C. cayetanensis* co-infection was higher among PLWH with CD4+ T cell counts below 200 cells/µL. Furthermore, individuals on cART with *C. cayetanensis* infection had been on cART for a significantly shorter duration compared to the group without this coccidian infection. These observations strongly indicate that co-infection is correlated with the level of immune suppression of the host. Importantly, co-infection with *C. cayetanensis*, a coccidian intestinal parasite, was associated with clinical symptoms, namely diarrhea over the previous 6 months and marked weight loss among those infected, which is in line with previous observations [7]. These findings are also broadly consistent with the body of literature demonstrating an association between immunosuppression and the risk of diarrhea-causing co-infection acquisition [28,29,30]. Intriguingly, however, the use of co-trimoxazole was not associated with significantly lower odds of co-infection; the adjusted odds ratio was 0.69 (95% CI: 0.35–1.29). In Ghana, as in many low-income countries, prophylactic use of co-trimoxazole, which shows antimicrobial activity on *C. cayetanensis*, is withdrawn among PLWH whose CD4+ T cell counts are above 200 cells/µL after initiation of cART in accordance with WHO guidelines [31]. A study of HIV-positive patients in Haiti found that symptomatic infection recurred in 43% who were followed up for more than one month after an initial 10-day course of co-trimoxazole treatment [26].

Apart from the known associations between low CD4+ T lymphocyte cell count and risk of acquisition of *C. cayetanensis* among PLWH, we provide new information on the association of this parasite with markers of immune activation, cell proliferation, terminal differentiation, and exhaustion of CD4+ and CD8+ T lymphocyte sub-populations stratified for cART status. There were no significant differences observed in the markers of cellular proliferation (Ki67) and terminal differentiation (CD57+) between those with and without *C. cayetanensis* co-infection. However, PLWH with *C. cayetanensis* co-infection exhibited a distinct immune profile, and thereby, CD4+ and CD8+ T-lymphocytes significantly expressed elevated markers of unfavorable immune activation (HLA-DR+CD38+) and CD8+ T-lymphocytes sub-populations demonstrated significant immune exhaustion (PD-1). The expression of the Programmed Death-1 (PD-1) marker, an inducible molecule on lymphocytes that portends high susceptibility to apoptosis and negatively regulates T cell activity, may be required to prevent the acquisition of diarrhea-causing coccidian parasites such *C. cayetanensis*.

### 4.1. Implications of the Study Findings

It has been advocated that regular 6-monthly screening of PLWH with low CD4+ T cell counts for the presence of stool parasites may be a cost-effective approach for targeted therapy against diarrhea. Patients may be non-adherent to co-trimoxazole, especially when cART is initiated, and are prone to co-infections with parasites such as *C. cayetanensis*, especially in the setting of sub-optimal immune reconstitution. This is important, especially among those on cART who are virologically suppressed but have low CD4+ T cell counts. As this study has a cross-sectional design, any causal relationship between the presence of *C. cayetanensis* and elevated immune activation parameters cannot be definitely confirmed. However, as shown in our study, patients colonized with *C. cayetanensis* suffer more often from diarrheal disease and weight loss and present with a distinctly unfavorable immune profile, so a prolongated chemoprophylaxis with co-trimoxazole in cases with clinical symptoms may be considered.

### 4.2. Limitations of the Study

A recent study comparing PCR assays for the detection of *Cyclospora cayetanensis* in stool samples demonstrated differences in the sensitivity of the tests evaluated [19]. Consequently, comparisons of detection rates across studies using different diagnostic methods should be made cautiously. Nevertheless, all methods evaluated have shown good specificity making a significant proportion of false-positive results rather unlikely. Another point that should be kept in mind is the seasonality of *C. cayetanensis* [32]. However, as all subgroups in our study population were included over the period of one year in a parallel design, seasonal fluctuations should have impacted all subgroups, and therefore, the risk for bias for the group comparisons is graded as low. Another limitation of the present study is that we did not record the duration of individual co-trimoxazole prophylaxis. Recurrence is common after short treatment periods. This missing information might have brought additional insights.

## 5. Conclusions

In the modern post-cART era, acquisition of *C. cayetanensis* among PLWH in Ghana is largely associated with an immunosuppression profile characterized by high expression of markers of immune activation and immune exhaustion.

## Figures and Tables

**Table 1:** Demographical and socioeconomic parameters as well as medical treatment in HIV-infected individuals according to *C. cayetanensis* status.

	Variable	HIV-Positive *C. cayetanensis* Positive, *n* = 56 (8.75%)	HIV-Positive *C. cayetanensis* Negative, *n* = 584 (91.25%)	*p*-Value
Demographics	Age in years ± SD	39.0 ± 9.2	40.7 ± 9.5	0.208
	Female, *n* (%)	37 (67.27)	425 (74.43)	0.321
Socioeconomic parameters	Access to tap water, *n* (%)	30 (54.55)	303 (53.06)	0.945
	Electricity in household, *n* (%)	54 (98.18)	531 (92.99)	0.230
	Television in household, *n* (%)	47 (85.45)	464 (81.26)	0.559
	Refrigerator in household, *n* (%)	39 (70.91)	415 (72.68)	0.902
	Owning a car, *n* (%)	4 (7.27)	56 (9.81)	0.809
Medical therapy	Co-trimoxazole prophylaxis, *n* (%)	14 (26.92)	189 (33.69)	0.402
	Antiretroviral therapy, *n* (%)	19 (34.55)	242 (42.38)	0.326
	Months since initiation of cART, median (IQR)	17.3 (6.9–36.6)	55.2 (28.6–79.5)	0.001

SD—standard deviation; cART—combined antiretroviral therapy.

**Table 2:** Virological and immunological parameters according to *C. cayetanensis* status in all HIV-positive patients and stratified by cART exposure.

Variable	HIV-Positive Cohort			HIV-Positive cART Exposed			HIV Positive cART Naïve		
	*C. cayetanensis* Positive, Median (IQR), *n* = 56	*C. cayetanensis* Negative, Median (IQR), *n* = 584	*p*-Value	*C. cayetanensis* Positive, Median (IQR), *n* = 19	*C. cayetanensis* Negative, Median (IQR), *n* = 242	*p*-Value	*C. cayetanensis* Positive, Median (IQR), *n* = 36	*C. cayetanensis* Negative, Median (IQR), *n* = 329	*p*-Value
Viral load, log10 copies/mL	5.0 (2.2–5.6)	4.1 (1.6–5.3)	0.012	1.6 (0.0–1.9)	1.6 (0.0–1.9)	0.126	5.4 (4.8–5.8)	5.1 (4.3–5.6)	0.054
CD4+ T-cell count/µL	228.0 (91.5–395.5)	355.0 (163.5–586.5)	0.001	308.0 (259.5–599.5)	516.5 (329.0–712.8)	0.053	141.0 (46.0–297.8)	246.0 (94.0–451.0)	0.012
CD8+ T-cell count/µL	938.0 (565.5–1279.0)	977.0 (659.5–1390.5)	0.576	896.0 (796.0–1172.0)	961.0 (656.2–1331.2)	0.961	988.0 (493.5–1388.0)	995.0 (661.5–1507.5)	0.477
CD4+/CD8+ T-cell ratio	0.3 (0.1–0.4)	0.4 (0.2–0.7)	0.005	0.4 (0.3–0.6)	0.5 (0.4–0.9)	0.205	0.2 (0.1–0.3)	0.3 (0.1–0.5)	0.013
HLA-DR+ CD38+ CD4+ (%)	31.0 (19.2–38.6)	17.3 (9.5–30.6)	0.002	16.4 (15.4–26.1)	10.5 (6.5–18.2)	0.018	33.4 (23.9–38.7)	23.6 (13.7–35.9)	0.043
HLA-DR+ CD38+ CD8+ (%)	49.3 (42.8–60.9)	43.4 (27.8–56.7)	0.042	45.6 (34.4–52.3)	27.5 (19.4–38.1)	0.045	54.0 (43.3–62.7)	51.5 (41.3–64.7)	0.565
CD57+ CD4+ (%)	19.2 (9.2–30.3)	14.4 (8.8–23.9)	0.293	16.9 (13.1–30.9)	13.3 (8.5–20.7)	0.274	19.5 (8.5–25.3)	14.4 (9.1–26.5)	0.726
CD57+ CD8+ (%)	46.9 (33.8–63.2)	48.4 (38.4–57.3)	0.816	39.0 (30.9–63.8)	51.2 (43.2–58.5)	0.227	52.9 (34.7–59.7)	44.8 (35.5–55.6)	0.480
PD-1+ CD4+ (%)	42.3 (29.8–63.2)	32.4 (22.6–46.1)	0.069	40.6 (22.3–53.8)	30.0 (18.7–39.9)	0.227	42.3 (30.2–66.1)	36.5 (24.6–52.8)	0.201
PD-1+ CD8+ (%)	43.1 (30.1–49.2)	31.9 (21.4–44.0)	0.042	38.0 (26.1–49.3)	25.0 (15.5–36.0)	0.074	44.6 (35.1–47.0)	38.7 (26.0–50.3)	0.334
Ki67+ CD4+ (%)	23.8 (14.7–26.8)	13.0 (6.3–30.6)	0.174	15.3 (10.7–21.1)	7.9 (5.1–16.4)	0.404	25.1 (17.0–42.3)	21.1 (9.6–44.7)	0.492
Ki67+ CD8+ (%)	16.0 (10.9–19.4)	10.2 (6.2–17.2)	0.153	21.7 (21.7–21.7)	6.7 (4.0–12.2)	0.219	14.0 (10.3–17.8)	12.0 (8.1–18.7)	0.658

cART—combined antiretroviral therapy; IQR—interquartile range.

## Data Availability

All relevant data are provided in the manuscript. Raw data can be made available upon reasonable request.

## Supplementary Materials

The following supporting information can be downloaded at: https://www.mdpi.com/article/10.3390/microorganisms10071407/s1, Figure S1: (a) Multiple logistic regression model displaying parameters associated with *C. cayetanensis* carriage in HIV-positive participants. (b) Multiple linear regression model displaying parameters associated with an increase in HLA-DR+ CD38+ CD4+ (%) in HIV-positive participants.
