# Supplementary material for: The Clinical Features and Immunological Signature of Cyclospora cayetanensis Co-Infection among People Living with HIV in Ghana
Source: Microorganisms. 2022 Jul 13;10(7):1407. doi: 10.3390/microorganisms10071407 (PMC9316080; doi:10.3390/microorganisms10071407)

Supplementary Figure S1

(a) Multiple logistic regression model displaying parameters associated with *C. cayetanensis* carriage in HIV positive participants. (b) Multiple linear regression model displaying parameters associated with an increase of HLA-DR+CD38+CD4+ (%) in HIV positive participants. Due to graphical restrictions of the 'forestmodel' package in R, the term "*C. cayentanensis*" is displayed in plain letters.

a. Factors associated with C. cayetanensis status

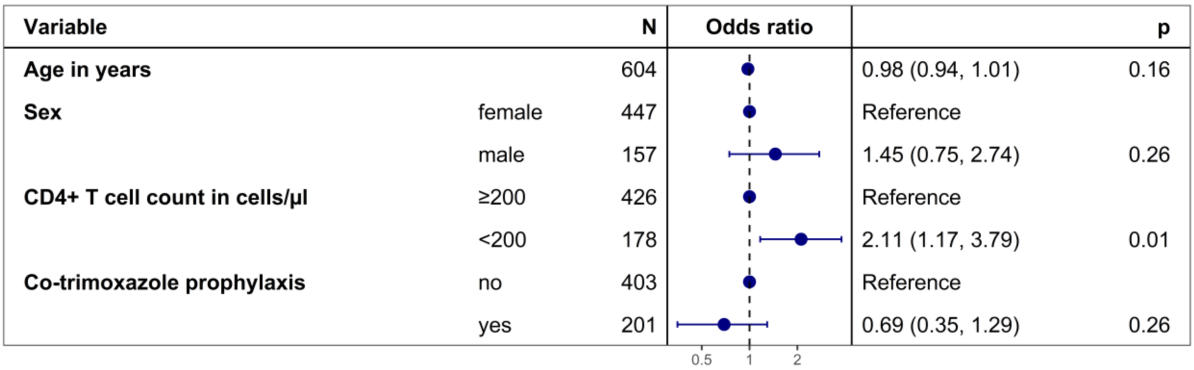

b. Factors associated with HLA-DR+CD38+CD4+ (%)

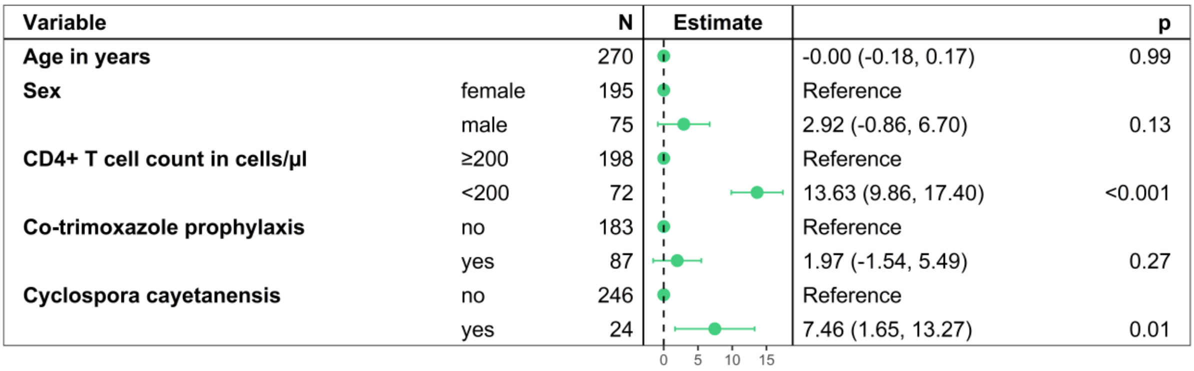

Supplement: Supplementary file 1 [file microorganisms-10-01407-s001.zip › microorganisms-1772675-supplementary.pdf]
